# Setting the pace: insights and advancements gained while preparing for an FES bike race

**DOI:** 10.1186/s12984-017-0326-y

**Published:** 2017-11-17

**Authors:** John McDaniel, Lisa M. Lombardo, Kevin M. Foglyano, Paul D. Marasco, Ronald J. Triolo

**Affiliations:** 1 Advanced Platform Technology Center, Louis Stokes Cleveland Veterans Affairs Medical Center, 10701 East Boulevard, Cleveland, OH 44106 USA; 2 Exercise Science Program, Kent State University, 350 Midway Drive, Kent, OH 44242 USA; 3 Laboratory for Bionic Integration, Department of Biomedical Engineering, Lerner Research Institute, Cleveland Clinic, 9500 Ave Cleveland, Euclid, OH 44195 USA; 4 Orthopedics and Biomedical Engineering, Case Western Reserve University, 10900 Ave Cleveland, Euclid, OH 44106 USA

**Keywords:** Spinal cord injury, CYBATHLON, Functional electrical stimulation, Cycling; exercise

## Abstract

The reduction in physical activity following a spinal cord injury often leads to a decline in mental and physical health. Developing an exercise program that is effective and enjoyable is paramount for this population. Although functional electrical stimulation (FES) stationary cycling has been utilized in rehabilitation settings, implementing an overground cycling program for those with spinal cord injuries has greater technical challenges. Recently our laboratory team focused on training five individuals with compete spinal cord injuries utilizing an implanted pulse generator for an overground FES bike race in CYBATHLON 2016 held in Zurich, Switzerland. The advancements in muscle strength and endurance and ultimately cycling power our pilots made during this training period not only helped propel our competing pilot to win gold at the CYBATHLON 2016, but allowed our pilots to ride their bikes outside within their communities. Such a positive outcome has encouraged us to put effort into developing more widespread use of FES overground cycling as a rehabilitative tool for those with spinal cord injuries. This commentary will describe our approach to the CYBATHLON 2016 including technological advancements, bike design and the training program.

## Background

It is estimated that there are 282,000 individuals currently living with spinal cord injury (SCI) in the United States, with 17,000 new cases every year. Following their injury these individuals experience very unique physical, social and psychological changes resulting from decreased ability to perform activities of daily living and exercise. This often leads to secondary complications including: musculoskeletal decline, bone and joint disease, heart disease, altered lipid profiles, arterial circulatory insufficiency, clotting disorders and more; for a review see [1]. In addition, those with SCI are also at greater risk for poor mental health including depression [2]. Finding a means to implement exercise in this population is paramount, but has been limited to specialized gyms and restricted to upper body exercise. Stationary functional electrical stimulation (FES) cycling systems are commercially available and have been utilized as an exercise modality. To date the prominent mode of outdoor recreational cycling for those with SCI is hand cycling with a smaller percentage of the population utilizing a hybrid arm-leg FES cycling and more recently a commercially available mobile recumbent bike was introduced to the market in 2005 by Hasomed (Magdeburg, Germany). However the technical challenges and efficacy of stimulation-powered overground cycling have yet to be fully resolved. For example, the low peak powers produced with FES cycling (approximately 25 watts) are not enough to overcome rough surfaces, slight inclines or headwinds that are often encountered during outdoors cycling. Participation in the FES bike race at the CYBATHLON 2016 in Zurich, Switzerland catalyzed a renewed interest and motivation within our laboratory team to develop an overground cycling program for individuals with SCI. Over the course of this commentary we will describe the obstacles, tangible outcomes and reflections from participating in this event.

## Main text

### Our research program

Our neuroprosthetic research program focuses almost exclusively on providing options for individuals with paralysis following spinal cord injury (SCI), stroke or multiple sclerosis (MS) to independently perform functional activities of daily living, such as standing to retrieve objects from overhead [3], transferring between seating surfaces of different heights [4], stabilizing the trunk and pelvis to improve wheelchair propulsion efficiency [5] and facilitate bimanual reaching [6] and stepping short distances in the vicinity of the wheelchair to negotiate obstacles such as curbs and steps [7]. To achieve these functional goals we developed multichannel implantable pulse generators (IPGs) and muscle- and nerve-based electrodes to efficiently and effectively excite the peripheral nerves to generate repeatable, strong and isolated contractions of the major muscles of the lower extremities, pelvis and trunk. These surgically installed IPGs can deliver 8, 12 or 16 independent channels of biphasic, charge-balanced current controlled stimulation of varying amplitude (0.1–20 mA), frequency (1–50 Hz) and pulse duration (1–255 μsec) depending on the application. Power and command information for various activation patterns are transmitted wirelessly to the implants by a wearable external control unit (ECU) via a transcutaneous inductive link formed between a coil taped to the skin above the IPG and the implant. The implanted system contains no batteries, and the rechargeable ECU can provide of 4–12 h of continuous stimulation per charge (depending on stimulation parameters), and has easily removable commercially available Canon 7.2V1800mAh Li-ion batteries allowing the users to have multiple charged batteries ready for use.

These systems are for investigational use and are not available commercially. Details of the implanted and external components, surgical procedures for installation, and clinical results from exercise, rehabilitation and home use of the systems are described elsewhere [8, 9]. At the present time, the neuroprostheses have been implanted in more than 30 individuals with SCI or stroke. The long-term follow up, averaging 7 years post-discharge to home with the systems, shows good component reliability, stability of stimulated responses, continued patterns of usage, and maintenance of functional abilities enabled with stimulation [10]. The design of these systems and their clinical implementation were focused on making their users as functionally independent and self-contained as possible within the constraints of the research programs designed to explore sitting and standing balance, stepping and seated reach under which they were implanted. None were optimized with electrodes targeted at muscles specifically required for cycling.

### Considering participation in the CYBATHLON 2016

Exercise was always a necessary means to achieve the functional goals of our program, but any recreational uses of the implanted neuroprostheses were left up to imaginations of individual recipients. Historically our reconditioning exercise programs were designed to rebuild the strength and endurance and consisted of conventional high load, low repetition progressive resistance strengthening, and high repetition, low load endurance building protocols. Recipients sometimes grew bored of the mundane routines which often resulted in reduced compliance and ultimately measurable declines in functional performance. Stimulation used for recreational exercise that recipients actually looked forward to, rather than simply tolerated, did not factor into our implementation strategy – until the CYBATHLON challenged us to change our thinking.

As we considered participating in the games, we realized that we had all of the resources required to mount a competitive entry in the stimulation-driven bike race. Up to that time we had never seriously considered overground biking as an option for our implant recipients. Over the years many of our volunteers had previous experiences with commercially available stationary surface stimulation exercise bikes, and they wanted to continue using them after implantation. As a result we had expended considerable effort to interface our implanted technology with those devices, and succeeded in utilizing the on-board systems for controlling resistance, modulating stimulation and activating motorized assistance to issue appropriate commands to our IPGs, which enabled recipients to pedal the stationary ergometers with their implanted systems. Although this experience may have helped prepared us for the CYBATHLON, for our laboratory team overground biking was a new and unfamiliar undertaking. We were well aware of the decades of research and commercial efforts around the world dedicated to overground biking with surface stimulation [11, 12] and spinal nerve root stimulation [13]. We were at first a bit intimidated by the elegance of the sophisticated control systems, biomechanical modeling, and studies of physiological responses of SCI subjects to stimulation-powered biking that had been published. But we began in earnest by reviewing the literature and studying what other groups had done to inform our approach.

### The race-ready bike

**Fig. 1:**
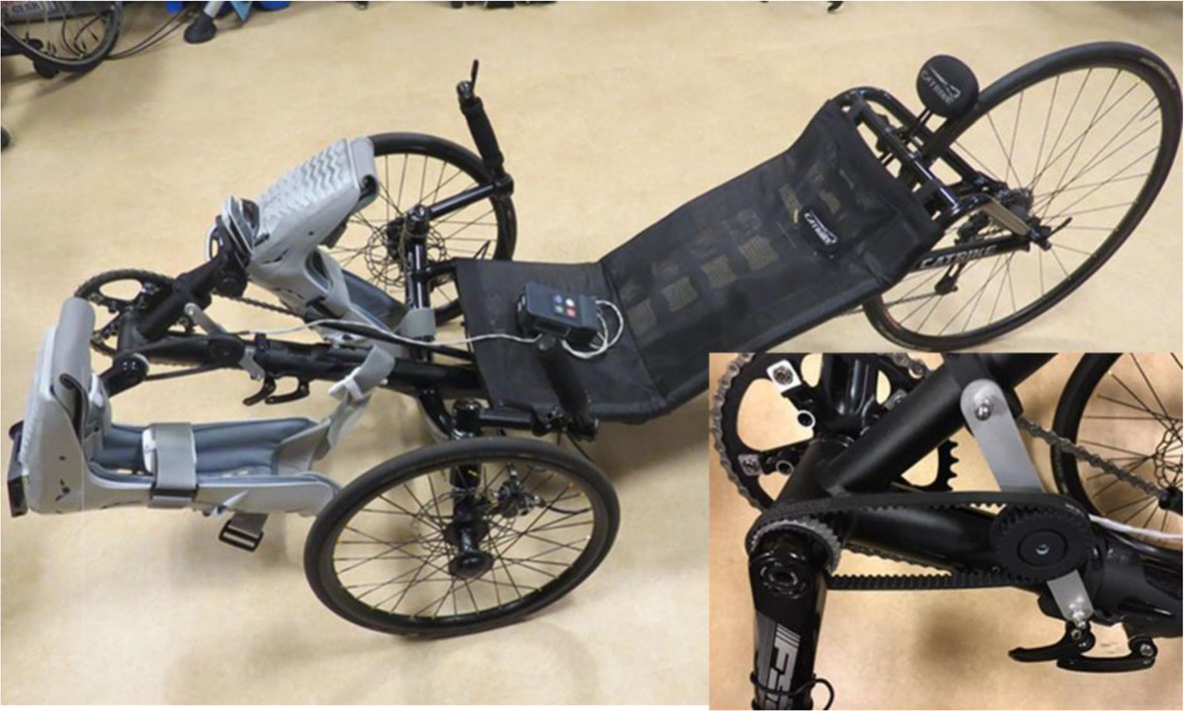
Picture of the modified trike with ECU on the seat. Note the ankle immobilizers were utilized to lock out the ankle. The insert illustrates the gear on the crank arm which is attached to the encoder that provides crank position to the ECU

We approached the CYBATHLON event as a serious competitive race and spent nearly just as much time focusing on the bike itself as we did the training program. Commercially available Catrike 700 recumbent tricycles were chosen as the race platform (Fig. 1). These tricycles have an excellent reputation in the adaptive cycling community and the machine itself was designed for performance cycling. The bikes required a minimum of specialized parts and the aluminum frames were light and allowed for modification and machining or repair if damaged. The only neural interface specific modification to the tricycles was the addition of a US digital MA3-A10–236-N Miniature Absolute Magnetic Shaft Encoder (with a resolution of 0.35 degrees) and bracket that monitored crank angle (Fig. 1 insert). The encoder gear was machined from aluminum and fit on the shouldered splines of the bottom bracket spindle. Crank angle information was sampled by the ECU, which modulated activation of the knee and hip extensor muscles appropriately to complete the pedaling motion. In all other aspects the tricycle was a normal bike.

The primary preparation of the tricycles for racing was focused on reducing weight and pedaling resistance. The hand-built rear wheel was provided by Topolino Technology (Bethel, CT). It consisted of a carbon hub shell with aluminum freehub body laced to a 700c alloy rim with carbon/kevlar composite spokes and aluminum alloy nipples; resulting in a slightly less than 800 g wheel. The front wheels were stripped down and re-laced with Sapim (Antwerp, Belgium) CX-Ray spokes and 7000 series alloy nipples. Folding-clincher Schwalbe (Ferndale, WA) Pro One and Continental Grand Prix TT tires were chosen to reduce weight and rolling resistance and coupled with ultralight butyl rubber tubes instead of latex tubes to prevent air loss overnight prior to the race. The carbon arm TRP Spyre (Ogden, UT) SLC brake calipers were coupled to ultralight Ashima (Taichung City, Taiwan) Ai2 brake rotors with both wheel brakes routed to a single Paul Component Engineering (Chico, CA) duplex brake lever. This allowed the pilot to brake both wheels simultaneously with the left hand thereby freeing the right hand to manipulate the command buttons on the ECU to start and stop stimulation. All extraneous parts of the tricycles were removed including non-essential bearing seals, springs, pads, seat storage, padding, chainrings, front derailleurs, left shifters, and chainring protectors. Adjustable booms to hold the crankarms were shortened to account for rider height. Due to these modifications the weight of the primary racing bike was reduced from the stock 13.6 kg (30 lbs) to 11.8 kg (26 lbs) and resulted in noticeable time improvements in several pilots during their training sessions (approximately 21 s over the 700 m course). 

Although the excessive time and energy spent crafting race-ready trikes may seem over ambitious for a 750 m flat course, we realized that if we could minimize the rolling resistance and make the bikes as light as possible, our pilots would have a better chance of cycling over the slight inclines in their neighborhoods and parks. We wanted to have a fast bike for the CYBATHLON, but we also wanted to maximize the capability for our pilots to ride the bikes outdoors. The race was the endpoint, but the effort also served the goals of enabling our pilots to exercise and engage in recreational activities independently in their homes and communities.

### The training program

**Fig. 2:**
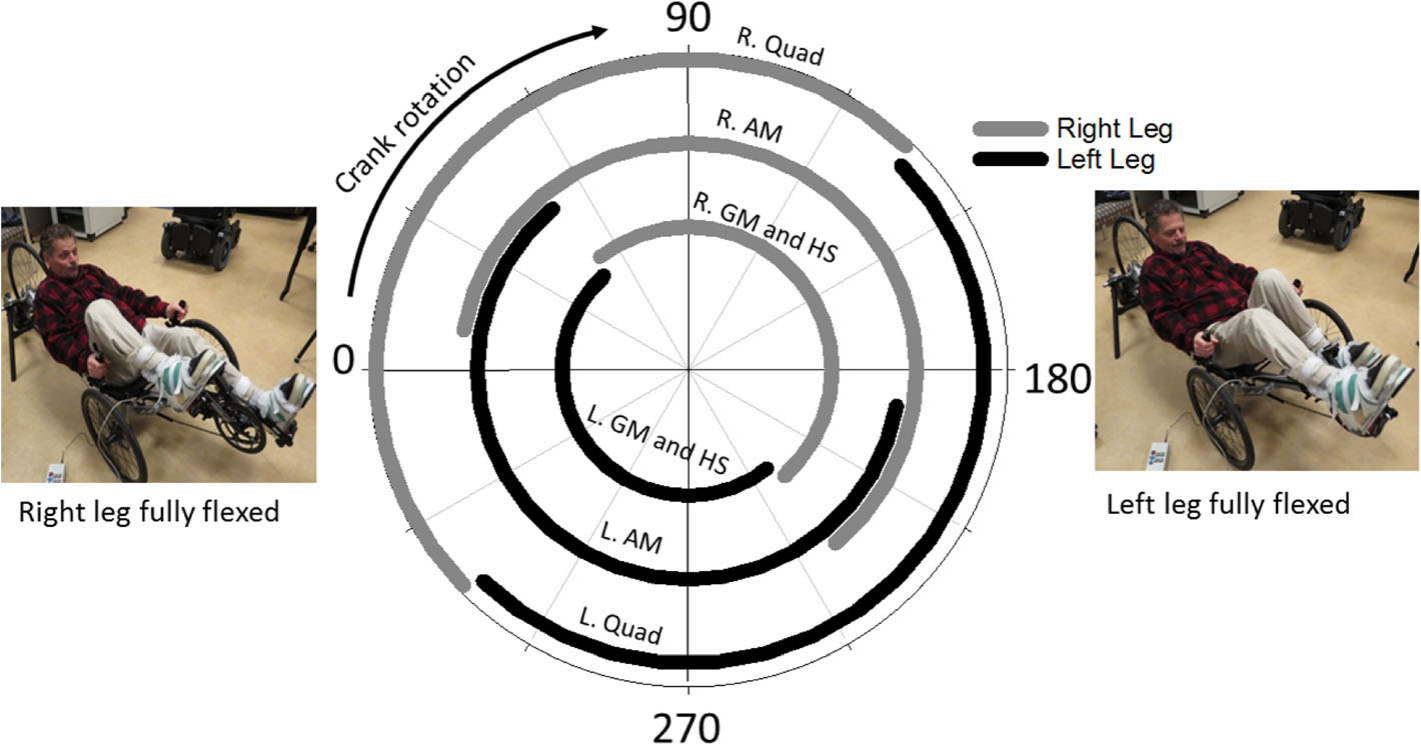
Stimulation patterns for the 5 pilots trained during this project. 0 degrees labels the position in which the right leg is fully flexed and begins to extend in the clockwise direction (as indicated by the black arrow). 180 degrees labels the position in left leg is fully flexed and begins to extend. Muscle abbreviations: R and L refer to right and left, Quad- quadriceps muscle group, AM- posterior portion of adductor magnus, GM- gluteus maximus, HS- hamstrings (with an emphasis on semimembranosus)

Prior to the development of a training program, we spent considerable time optimizing the stimulation parameters, body positioning and gear ratios for five pilots (Table 1) that would spend several months training for the CYBATHLON. The cycle activation patterns were based on the able-bodied and surface stimulation cycling literature [14], and then tailored to the implanted muscle sets for each pilot. Fine tuning of stimulus timing and pulse parameters continued until smooth pedaling motions were achieved without any dead spots. In general, the muscles stimulated with the implants for all 5 pilots were the gluteus maximus, posterior portion of adductor magnus, quadriceps muscle group and semimembranosus. Ultimately all five pilots utilized similar stimulation patterns (Fig. 2). Although there was no overlap in stimulation between the right and left quadriceps, gluteus or semimembranosus, there was approximately 40 degrees of stimulation overlap between the right and left adductor magnus primarily to minimize hip abduction. To protect the insensate joints and reduce potential loss of power due to excess lateral movement and external rotation of the legs, many different combinations of body positioning, upper body stabilization straps and foot/ankle attachments were tested. Ultimately, commercially available Aircast ankle-foot immobilizers were rigidly affixed to standard platform pedals just below the malleoli to optimize force transfer and constrain non-sagittal hip motion. These adjustments were primarily based on visual inspection of the cycling movement and feedback from the pilots. Furthermore our five pilots could transfer into and out of the Catrike independently and safe mastery of this transfer was part of our rehab/training program before sending them home with a bike.

**Table 1:** Subject characteristics

Subject	Gender	Age	Level of injury	Time since injury	Duration of implant	Previous physical activity level
S1	Female	45	C7 AIS B	18.4 years	16.7 years	Exercising and standing with FES
S2	Male	55	T4 AIS A	32.7 years	32.1 years	Exercising and standing with FES
S3	Male	51	T3 AIS A	3.3 years	1.6 years	Exercising with FES
S4	Male	56	T11 AIS B	7.5 years	5.2 years	Exercising and standing with FES
S5	Male	59	T4 AIS B	8.4 years	4.5 years	Exercising and standing with FES

Drivetrain gear ratios were determined based on pedaling rates and power production (Garmin Vector 2 powermeter pedals; Garmin International, Olathe Kansas) that could be achieved across all the cogs in a gear cluster (cassette) while the trike was on a stationary trainer. In the end, a 42 tooth front chainring coupled with a 15/16 tooth cog allowed the riders to produce a pedaling rate (approximately 40 rpms) that maximized the power produced during a 45 s bout of cycling. Thus an 11–23 10-speed cassette was fitted to the bike as it placed the 15/16 tooth cog at the center of the cassette range. This allowed them to adjust their gears up or down during the course of their training to account for environmental conditions (incline, wind, rough surfaces etc) and fatigue while the stimulation patterns and intensity remained constant. After these variables were optimized, all five pilots were sent home with a bike, stationary trainer and an ECU for their implanted system programed with specific stimulation parameters for predetermined cycling workouts.

**Fig. 3:**
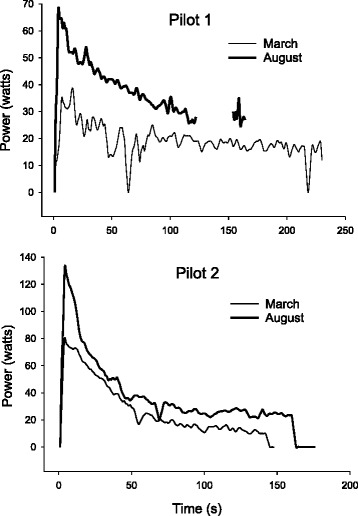
Power profile curves for two pilots across the training period. Note some data was dropped during the August test for pilot 1

Although our goal was to develop a training program based on our knowledge of the energy systems and the physiological adaptations we were hoping to obtain, we knew from the start that we would not be able to rely on traditional approaches to bicycle training. One major obstacle was the reverse order of muscle fiber recruitment (fast to slow) that occurs with electrical stimulation. This essentially eliminated the ability to include low intensity/ long duration days in our training program. Specifically, reducing the stimulation intensity would merely result in the primary recruitment of the fast twitch fibers which are more fatigable and therefore incompatible with a long duration training session. As a result all training sessions utilized stimulation intensities that maximized motor recruitment. After initial testing, we learned our pilots fatigued much quicker than anticipated, producing approximately 30 watts or more for 15–20 s before fatigue reduced power output to 10–15 watts for another minute which was insufficient to maintain overground propulsion velocity (Fig. 3). We also had to consider that without the influence of central command and an intact exercise pressor reflex, the pilots would not have normal cardiorespiratory responses from which we could gauge their effort.

Realizing the pilots initially fatigued very quickly, we developed a program that maximized the time they spent producing maximum power. The strength protocol consisted of 60 s of stimulation followed by 30 s of rest. They were instructed to perform this in the most difficult gear ratio that would still allow them to complete these intervals for one hour. A 1-h endurance protocol consisted of a 10-min bout of cycling followed by a 5-min rest. The pilots performed these protocols a total of 3–4 times per week on a stationary trainer with emphasis on the protocol that addressed their weakness (i.e., pilots with high power and low fatigue resistance focused on the endurance program and vice versa). Pilots were also instructed to keep diaries of their training logs and the ECU monitored compliance.

The pilots initially complied with the training program; however as they increased their strength and endurance they often diverged from the program, and for good reason. During the initial overground testing, we realized that the pilots would need to produce approximately 20–25 watts to simply maintain overground velocity on a flat surface, and at the time their quick rate of fatigue made overground cycling nearly impossible. As they became stronger and less fatigable, they realized they could maintain the necessary power to cycle overground for extended periods of time and subsequently preferred to ride outdoors in their neighborhoods or parks and leave the constraints of the stationary trainer. It was the first time that they were able to exercise outdoors on their own in the community and, based on feedback from the pilots, having the ability to ride outdoors had a strong impact on their motivation to train.

As we prepared for the CYBATHLON, another major focus was optimizing race day performance and recreating race conditions. Pilots simulated race conditions using a regulation size ramp to determine which gear to start with to take advantage of the early peak power output, to become efficient at shifting gears as they fatigued, and to practice switching lanes. During this time each pilot developed a sense of how much warm up time they needed for optimal performance. We also trained our pilots to mimic a normal respiratory response by increasing breathing frequency from the start.

**Fig. 4:**
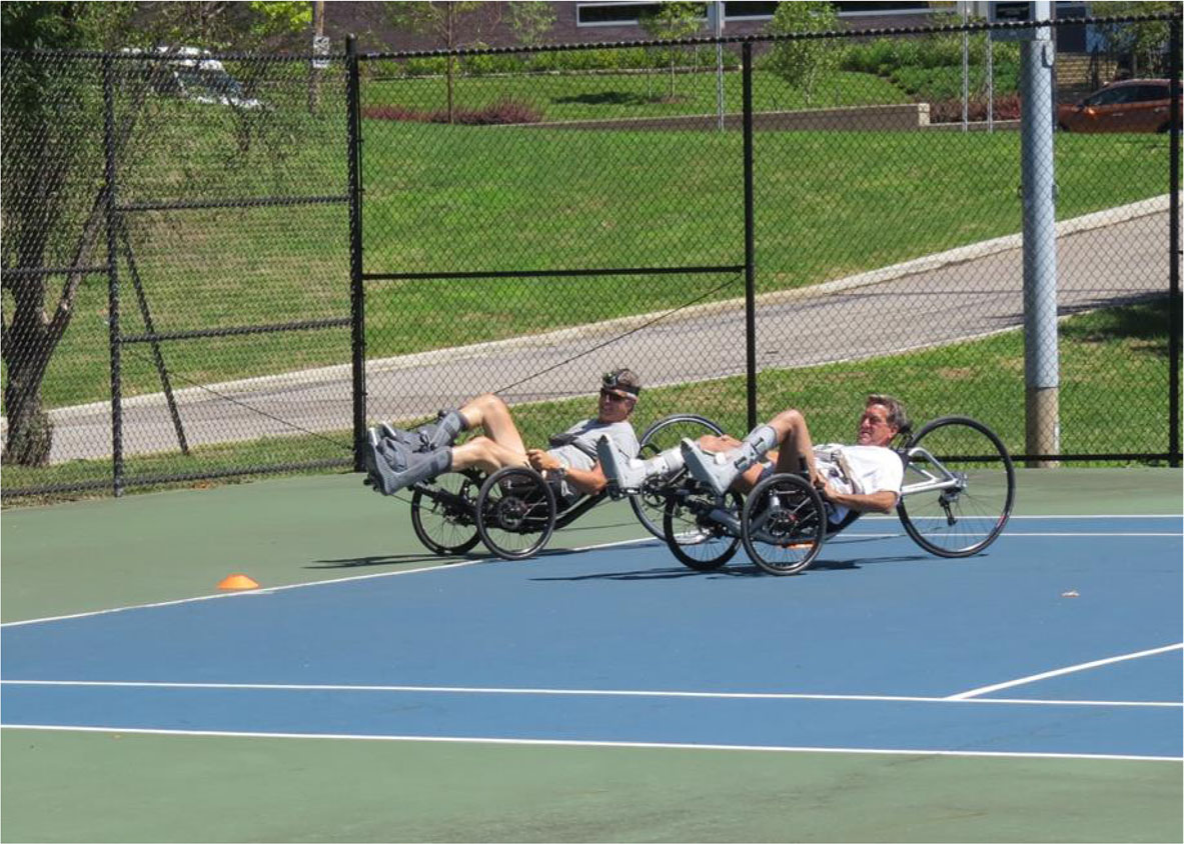
Two pilots performing a time trial prior to the CYBATHLON

**Fig. 5:**
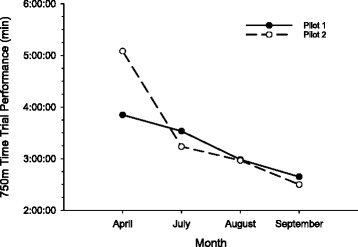
Improvements in 750 m time trial performance for two pilots

During the training period we routinely reexamined power and performed 750 m time trials to monitor improvement (Fig. 4). This was not only valuable for us as coaches and physiologists, but also provided a source of motivation for our pilots. In the end the training did result in substantial improvements in the power profile (Fig. 3) as well as the 750 m time trial performances (Fig. 5). Two months prior to the CYBATHLON we held a time trial to determine which two of our five pilots would go to Switzerland. During this event, all five pilots produced race times that would have at least advanced them from the qualifying rounds of the CYBATHLON and four of the five pilots would have advanced to the gold medal round.

### Addressing the elephant in the room

**Fig. 6:**
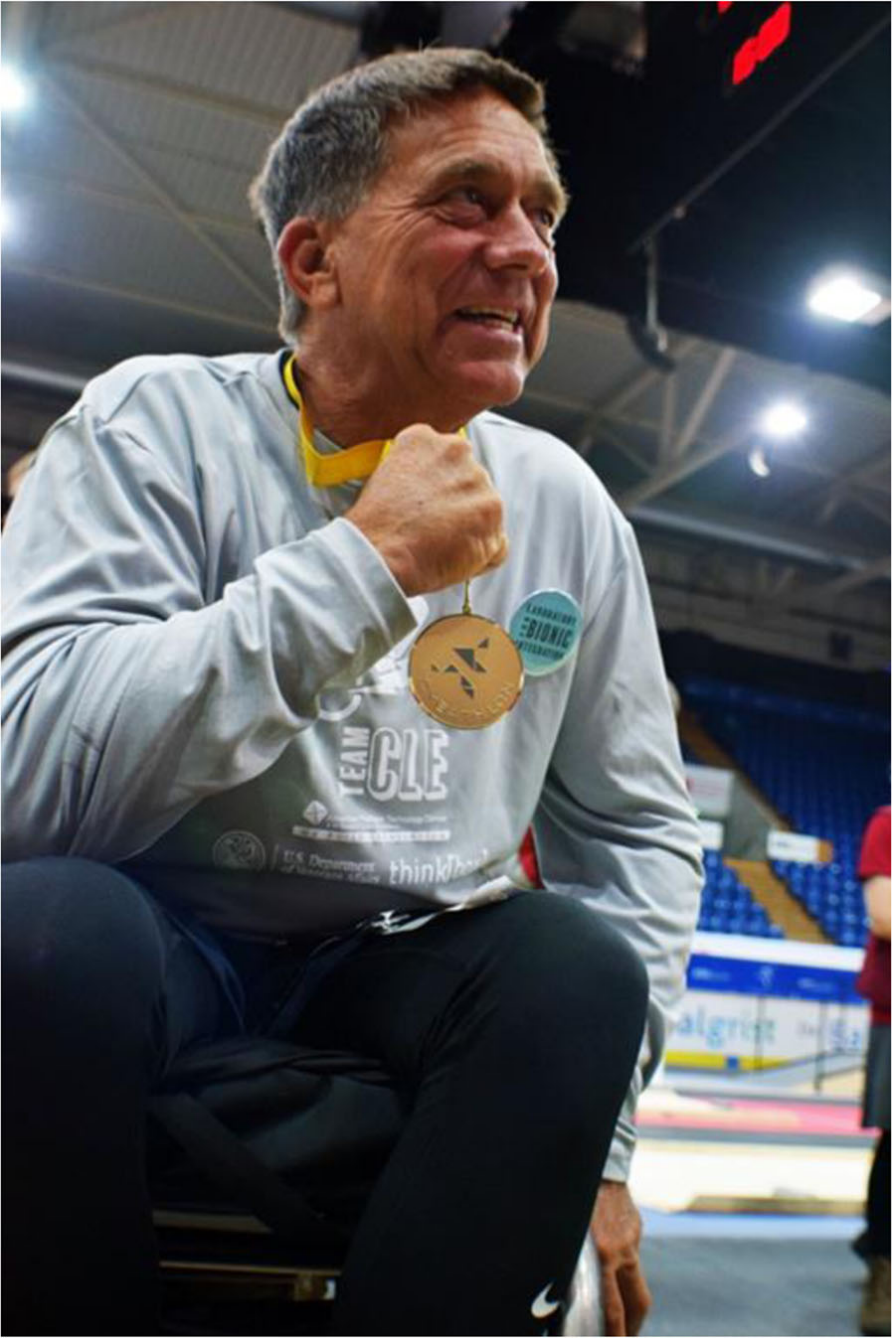
Picture of our pilot with the gold medal from the CYBATHLON

One major difference between our pilots and every other pilot participating in the CYBATHLON was the use of our implanted system that provided selective and repeatable activation of the targeted muscle groups. This undoubtedly gave our pilots an advantage over the competition, which all relied on surface stimulation, and helped propel our pilot to win the gold medal (Fig. 6). While it is impossible to quantify the relative magnitude of the benefits of our training and conditioning program, bicycle modifications, stimulation patterns or control strategy toward race performance retrospectively, the implanted system is likely the major contributor. The full extent of the advantages of the implanted system is probably masked by other factors, such as the relatively young ages of the pilots and long histories and wealth of experience of other groups in the competition with FES cycling compared to our pilot and team. In the future, the enhanced power output that appears to be possible with the implanted system may enable individuals who are not currently candidates for overground cycles, such as those with significant upper extremity impairments who cannot manipulate handcranks or with poor responses to surface stimulation, take full advantage of what cycling has to offer.

We fully recognize and acknowledge that utilization of surface stimulation is paramount to widespread dissemination and use of overground cycling by individuals with SCI, perhaps even in preparation for receiving an implanted system, and have recently started to more thoroughly compare the efficacy of using FES cycling with surface or implanted electrodes. The month after competing in the CYBATHLON, our race pilot performed a trial of cycling with surface electrodes in our laboratory. The power he was able to produce was approximately 25% lower with surface stimulation than with the implanted system. It is unclear whether this is an accurate assessment of the performance of each stimulus delivery system, since a portion of the muscle fibers excited by surface stimulation may be different from those recruited by the implanted system, and therefore not optimally reconditioned. We are looking forward to continuing to explore and optimize the relative benefits of overground cycling with either surface or implanted systems so that more individuals with SCI can derive the health benefits from the exercise and recreational modality.

## Conclusion

### Reflection on the CYBATHLON 2016 experience

**Fig. 7:**
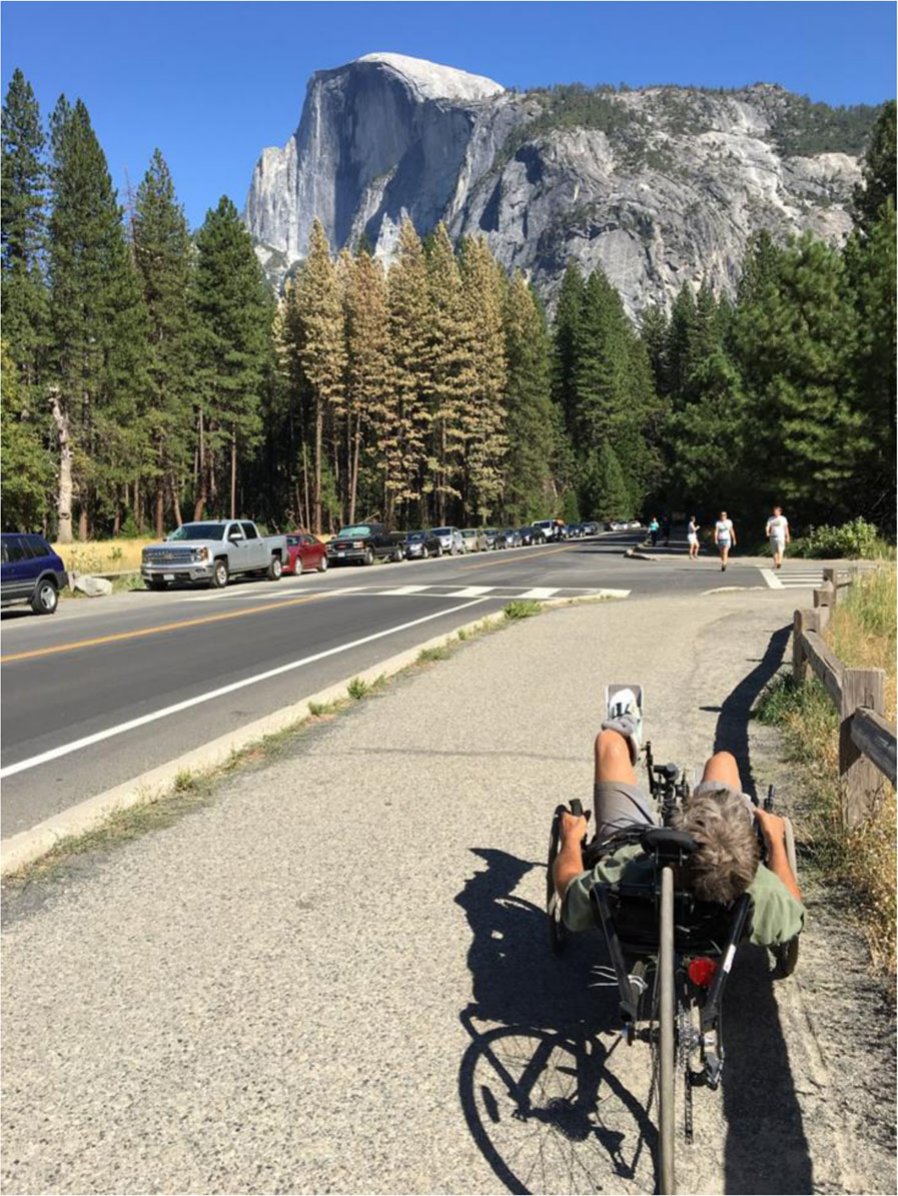
Picture of one of our pilots cycling near Half Dome in Yosemite National Park, California

In the setting of the CYBATHLON, we saw the bicycle as a machine that provides a tangible path to self-improvement and independence. The instant acceptance and use of the technology was a surprise to our research team members and pilots alike. In rehabilitation research, we are often faced with the realization that the techniques and technologies we develop will help move someone a step forward in their physical well-being and independent personal, professional or societal function, but the advances are small and hard-fought. In the case of implant-driven cycling after spinal cord injury, the payoff was tangible, immediate and profound. The benefits were demonstrable as the pilots rode the bikes under their own power and speed, without handcranks or motors, making it easy to forget they were paralyzed from the chest down. The competition galvanized our attention and enabled us to think creatively and work collaboratively with our pilots outside of the rigor of hypothesis driven research to achieve these goals. The most exciting aspect of the CYBATHLON cycling experience was that it provided a means for the pilots to take the systems home and train with them outside on their own. During this time they rode down streets to grab a cup of coffee, they rode with friends through their neighborhoods, and they even rode with their families in national parks (Fig. 7). In so doing, they rode a bit closer to health and independence, and we all rode closer to a deeper understanding of the potential of implanted assistive technologies.

## Abbreviations

ECU: External control unit
FES: Functional electrical stimulation
IPB: Implanted pulse generator
